# Molecular characterization of a Chinese variant of the Flury-LEP strain

**DOI:** 10.1186/1743-422X-7-80

**Published:** 2010-04-28

**Authors:** Linzhu Ren

**Affiliations:** 1 College of Animal Science and Veterinary Medicine, Jilin University, Changchun 130062, China

## Abstract

The entire genome of rabies virus vaccine strain Flury-LEP-C, a Chinese variant of the rabies virus vaccine strain Flury-LEP, was sequenced. The overall length of the genome of Flury-LEP-C strain was 11 924 nucleotides (nt), comprising a leader sequence of 58 nt, nucleoprotein (N) gene of 1353 nt, phosphoprotein (P) gene of 894 nt, matrix protein (M) gene of 609 nt, glycoprotein (G) gene of 1575 nt, RNA-dependent RNA polymerase (RdRp, L) gene of 6384 nt, and a trailer region of 70 nt. There was TGAAAAAAA (TGA_7_) consensus sequence in the end of each gene in Flury-LEP-C genome, except G gene which had a GAGAAAAAAA sequence in the end of the non-coding G-L region. There were AACAYYYCT consensus start signal close to the TGA_7_. Flury-LEP-C has 310 nucleotides more than HEP-Flury in G-L intergenic region. The analysis showed that the residue at 333 of the mature G protein was Arg, which was reported to be related to pathogenicity. Compared with FluryLEP, there were 19 different amino acids (AAs) in five proteins of Flury-LEP-C, including 15 AAs which were identical with corresponding residues of Hep-Flury, and 4 AAs which were neither identical with the residues of FluryLEP nor with the residues of Hep-Flury. The results showed the topology of the phylogenetic trees generated by two protein sequences were similar. It was demonstrated that HN10, BD06, FJ009, FJ008, D02, D01, F04, F02 have a close relationship to CTN-1 and CTN181, and MRV was closely related to Flury-LEP, HEP-Flury and Flury-LEP-C.

## Findings

The rabies virus belongs to the *Rhabdoviridae *family and the *Lyssavirus *genus. The genome of the rabies virus is a non-segmented, anti-sense, single-stranded RNA which is about 12, 000 nucleotides (nt) long. Viral RNA encodes five major proteins: nucleoprotein (N-protein), phosphoprotein (P protein), matrix protein (M-protein), glycoprotein (G-protein) and RNA-dependent RNA-polymerase (L-protein) [1].

It was reported there were still high rabies cases happened in China, especially in rural China, about 5537 fatalities per year in 80's, and about 3300 fatalities in 2007 [2-5]. During recent years, most of the research on the control of rabies has concentrated on the development of oral vaccine, including attenuated vaccine and live vectored vaccines. However, these virus strains are still pathogenic for laboratory and wild rodents or wildlife species, and several rabies cases caused by such vaccines have been reported [6,7]. It was reported some rabies virus in China was closely related to several vaccine strains [8]. The main goal of the present study was to obtain the entire genome sequence of vaccine strain Flury-LEP-C, a Chinese variant of the rabies virus vaccine strain Flury-LEP, including the 3'- and 5'-terminal non-coding regions of the genome. The genome sequence has been compared to the sequences of other vaccine strains used in China and street strains in China available from GenBank. The data obtained from vaccine strain and street strain can lead to a better understanding and more effective strategies to control the spread of rabies.

Here, we obtained the full length genome of Flury-LEP-C strain by RT-PCR or RACE similar to the method described by Marston et al. [9]. Using a total of 12 primers (as shown in Table 1), the entire genome of Flury-LEP-C strain was amplified as 5 separate overlapping PCR products. The result showed that the full genome of rabies virus strain Flury-LEP-C consists of 11924 nt. The full length sequence was submitted to GenBank (GenBank accession numbers FJ577895).

**Table 1:** Primers used for amplification of the Flury LEP strain

Designation	Sequence of primers	Length	Location
RLM-3' RACE oligonucleotides^a^	5'-GTCGTACTAGTCGACGCGTGGCCTAG-3'	26	
3' RACE complementary oligonucleotides^a^	5'-GGCCACGCGTCGACTAGTAC-3'	20	
LEPR1(antisense)	5'-CAAGAGGGCCCCTGGAATCA-3'	20	2872-2892
LEPF2(sense)	5'-TCCAGGGGCCCTCTTGAAGGGGAG-3'	24	2875-2899
LEPR2(antisense)	5'-ATGACCGGTCTTCACAGTCTGGTC-3'	24	4881-4905
LEPF3(sense)	5'-GTGAAGGCCGGTCATCCTTTTGACAATT-3'	28	4888-4916
LEPR3(antisense)	5'-CAAGAGACTCGGGCCCAT-3'	18	7836-7854
LEPF4(sense)	5'-GATGGGCCCGAGTCTCTTGC-3'	20	7833-7853
LEPR4(antisense)	5'-TAACACAAGATCGATCTGTTG-3'	21	9905-9926
LEPF5(sense)	5'-CCACTATGAAAGAAGGCAACAGATCGATC-3'	29	9888-9917
5' RACE Outer Primer	5'-CATGGCTACATGCTGACAGCCTA-3'	23	
5' RACE Inner Primer	5'-CGCGGATCCACAGCCTACTGATGATCAGTCGATG-3'	34	

^a^These two oligonucleotides were synthesized according to Marston et al. (2007).

**Figure 1:**
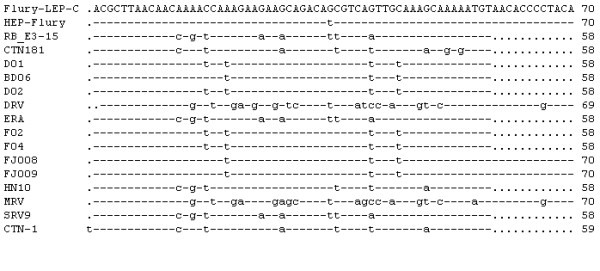
**Comparison of 3'-termini of the antigenome (+) sense RNA (in DNA code)**. 3'-termini of Flury-LEP-C strain and other rabies virus were compared. Only differences from the reference sequences are shown. "-" indicate sequence identity to the reference sequence and a "." indicate missing sequence.

**Figure 2:**
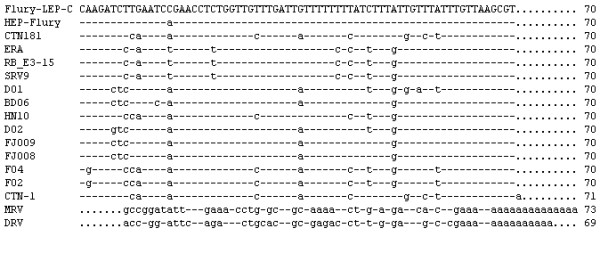
**Comparison of 5'-termini of the antigenome (+) sense RNA (in DNA code)**. 5'-termini of Flury-LEP-C strain and other rabies virus were compared. Only differences from the reference sequences are shown. "-" indicate sequence identity to the reference sequence and a "." indicate missing sequence.

In the full genome sequence of Flury-LEP-C, the leader sequence was 58 nt in length, while trailer sequence was 70 nt. All RVs (as shown Table 2) in this study were absolutely conserved over the 12 bases of the genomic 3'-terminus (Fig. 1) and 5'-terminus (Fig. 2). The sequences of 3' leader and 5' trailer termini showed exactly complementary for the terminal 11 nt of all RVs, except that MRV and DRV showed different 3'-terminus and 5'-terminus end.

**Table 2:** Rabies virus referenced in this study

Isolate Name	Strain Information	Accession numbers
Flury-LEP-C	vaccine strain maintained in BHK-21 cells; derived from the vaccine strain FluryLEP	FJ577895
FluryLEP	Vaccine strain	DQ099524
Hep-Flury	Vaccine strain derived from the vaccine strain FluryLEP	AB085828
SRV9	Avirulent vaccine strain maintained in BHK-21 cells	AF499686
CTN181	Isolated from rabies patient; vaccine Strains for Human use	EF564174
CTN-1	Isolated from the brain of rabies patient; vaccine strain for human use	FJ959397
RB/E3-15	A adapted vaccine strain maintained in Vero cells	EU182346
ERA	Attenuated rabies vaccine strain derived from SAD strain	EF206707
D02	Isolated from ferret badger and dog rabies	FJ712194
D01	Isolated from ferret badger and dog rabies	FJ712193
HN10	Isolated from the brain of rabies patient	EU643590
FJ009	Isolated from dog	FJ866836
FJ008	Isolated from dog	FJ866835
F04	Isolated from the brain of Chinese ferret badger	FJ712196
F02	Isolated from the brain of Chinese ferret badger	FJ712195
BD06	Street strain	EU549783
DRV	Street strain	DQ875051
MRV	Street strain	DQ875050

Between the transcription stop and start signals, there was an intergenic sequence (IGS), which was not transcribed into mRNA. The N/P IGS was CT. The P/M IGS was CAGGC, and M/G IGS was CTATT. The IGS between the non-coding G-L region and L gene was 21 nt.

**Figure 3:**
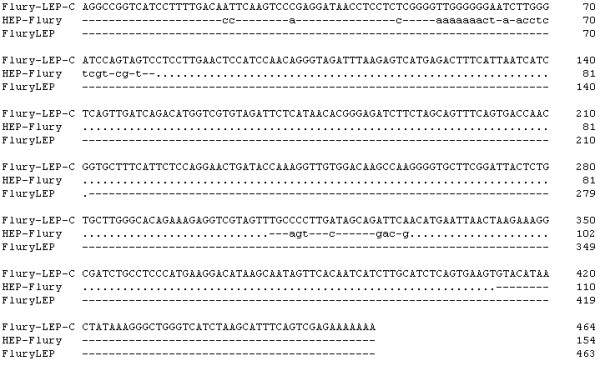
**Nucleotide acid sequence alignment of the non-coding G-L region of Flury-LEP-C, Flury LEP and HEP-Flury**. "-" indicate sequence identity to the reference sequence and a "." indicate missing sequence.

The G-L intergenic region is a non-coding region. It was reported that this region was highly susceptible to random mutations, unrestricted by structure and function requirements or by immunological pressure [10]. Comparison result in this study showed that the G-L intergenic region of Flury-LEP-C has 310 nucleotides more than that of HEP-Flury (Fig. 3), which demonstrate that the non-coding G-L region was more prone to mutate. The observation indicates that the region may be used as an insertion site for a marker gene to construct a marker vaccine. However, studies should be undertaken to confirm this hypothesis.

Rabies virus encodes five structural proteins in the order of N-P-M-G-L. The length of five genes of Flury-LEP-C strain were 1353 nt, 894 nt, 609 nt, 1575 nt, 6384 nt, respectively. There was TGAAAAAAA (TGA_7_) consensus sequence in the end of each gene in Flury-LEP-C genome, except that G gene had a GAGAAAAAAA sequence in the end of the non-coding G-L region. There were AACAYYYCT consensus start signal close to the TGA_7_. The main difference between Flury LEP and Flury-LEP-C was that the latter has 12 nt more than the former in L gene (Table 3). Further studies are necessary to elucidate the role of these mutations in Flury-LEP-C.

**Table 3:** Different proteins of Flury-LEP-C compared with FluryLEP and Hep-Flury strains.

Protein	FluryLEP	Flury-LEP-C	Hep-Flury
N	Y288	H288	H288
P	D59	G59	D59
	F115	L115	L115
	L186	W186	W186
	K231	E231	E231
M	A22	V22	A22
G	V13	G13	V13
	H283	R283	R283
	Q297	K297	K297
	E368	G368	E368
	Y371	H371	H371
	I415	M415	M415
	T510	I510	I510
L		Y166	Y166
		L167	L167
	N387	D387	D387
	I450	V450	V450
		N833	N833
		A834	A834

The entire amino acid sequence of Flury-LEP-C was aligned with 17 entire genome sequences (as shown in table 2) obtained from the GenBank. Analysis of deduced amino acid sequences from open reading frames (ORFs) of N, P, M, G, and L genes revealed 98.81%, 93.94%, 96.75%, 95.12%, 97.69%. Szanto reported that P gene was the most variable gene[11], similar result was obtained in Flury-LEP-C.

The G gene does indeed encode a product of 524 amino acids but this includes a 19 amino acid N-terminal signal peptide that is cleaved to generate the mature product of 505 amino acids. It was reported that the G protein plays an important role in viral pathogenicity and protective immunity, especially residue Arg333 [1,12-17]. Jackson et al. reported that less neurovirulent strain, which contains an attenuating substitution of Arg333 in the rabies virus glycoprotein, was a stronger inducer of neuronal apoptosis and there was an inverse relationship between pathogenicity and apoptosis [18]. In this study, the analysis showed that the residue at 333 of the mature G protein was Arg.

P protein is a structural component of the RNP. And P protein is also crucially involved in numerous events during the virus life cycle, including proper formation of viral RNPs and virus particles and viral RNA synthesis [14]. The P protein has been shown to interact with LC8 (cytoplasmic dynein light chain) at residues 138-172 [19,20], specifically the motif K/RXTQT at residues 145-149 [20]. Mebatsion found that the deletions introduced into the LC8 binding site abolished the P-LC8 interaction, blocked LC8 incorporation into virions, and reduced the efficiency of peripheral spread of the virus, but LC8 is dispensable for the spread of a pathogenic RV from a peripheral site to the CNS [19]. We found that the minimal binding motif for LC8 at residues 145-149 of P protein was KSTQT in all rabies sequences in this study, except that SHBRV-18 has a KATQT motif.

Compared with FluryLEP, there were 19 different amino acids (AAs) in five proteins of Flury-LEP-C, including 15 AAs which were identical with corresponding residues of Hep-Flury, and 4 AAs which were neither identical with the residues of FluryLEP nor with the residues of Hep-Flury (table 3). Comparison of L protein of all RVs in table 2 showed that all RVs, except Hep-Flury and FluryLEP, have these four insertions in L protein. Studies are undertaking to find difference in phenotypic characteristics between the Flury-LEP-C and its parental strain FluryLEP.

**Figure 4:**
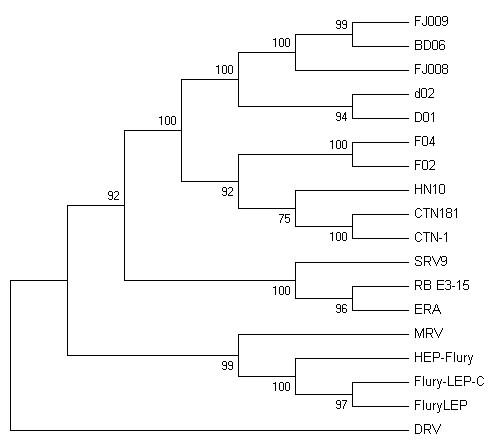
**Phylogenetic tree of 18 rabies viruses generated with coding sequences by a NJ analysis with the Kimura parameter**. Bootstrap values out of 1000 replicates are indicated as a percentage to the left of each branch of the tree. Nucleotide sequences of five viral genes of each strain were translated into protein sequences and joined to one sequence in the original order, based on which a phylogenetic tree was generate.

**Figure 5:**
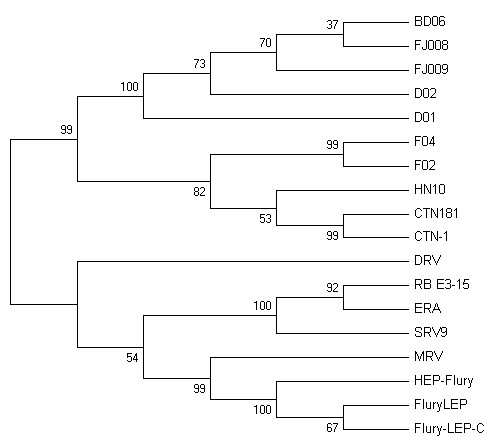
**Phylogenetic tree of 18 rabies viruses generated with amino acids sequences of P protein by a NJ analysis with the Kimura parameter**. Bootstrap values out of 1000 replicates are indicated as a percentage to the left of each branch of the tree. P proteins of all RVs were used to construct the phylogeny tree.

In this study, two kinds of proteins were used to construct the phylogeny tree. First, nucleotide sequences of five viral genes of each strain were translated into protein sequences and joined to one sequence in the original order, based on which a phylogenetic tree was generate (Fig. 4). Second, P protein, due to its multifunctional nature including its ability to interact with host-cell proteins [21], were also used to construct a phylogeny tree (Fig. 5). The results showed the topology of the phylogenetic trees generated by these two methods were similar. It was demonstrated that HN10, BD06, FJ009, FJ008, D02, D01, F04, F02 have a close relationship to CTN-1 and CTN181, which means the homology between the CTN stains and the Chinese street strains was much higher than that of any other vaccine strain. And MRV was closely related to Flury-LEP, HEP-Flury and Flury-LEP-C, but DRV formed an outlying clade. The CTN (or its derivates, including CTN-1 and CTN181), PV and PM strains are the human rabies virus vaccine strains, and FluryLEP, HEP-Flury, ERA and CTN-1 are the veterinary rabies virus vaccine strains currently used in China. It was hypothesized the CTN strain should be most suitable for use in China as a vaccine strain [10,22], and the result in our study also supported the hypothesis.

## List of abbreviations

RACE: rapid amplification of cDNA ends; RV: rabies virus; RT-PCR: Reverse transcription polymerase chain reaction; RNP: ribonucleoprotein.

## Competing interests

The author declares that they have no competing interests.

## Authors' contributions

The author has made substantial contributions to design, acquisition of data, analysis and interpretation of data, and draft the manuscript.
